# In-hospital mortality and failure to rescue following hepatobiliary surgery in Germany - a nationwide analysis

**DOI:** 10.1186/s12893-020-00817-5

**Published:** 2020-07-29

**Authors:** Christian Krautz, Christine Gall, Olaf Gefeller, Ulrike Nimptsch, Thomas Mansky, Maximilian Brunner, Georg F. Weber, Robert Grützmann, Stephan Kersting

**Affiliations:** 1 Universitätsklinikum Erlangen, Friedrich-Alexander-Universität Erlangen-Nürnberg, Klinik für Allgemein- und Viszeralchirurgie, Krankenhausstraße 12, 91054 Erlangen, Germany; 2 Friedrich-Alexander-Universität Erlangen-Nürnberg, Institut für Medizininformatik, Biometrie und Epidemiologie, Waldstraße 6, 91054 Erlangen, Germany; 3 Technische Universität Berlin, Department of Health Care Management, Straße des 17. Juni 135, 10623 Berlin, Germany; 4 Technische Universität Berlin, Department for Structural Advancement and Quality Management in Health Care, Straße des 17. Juni 135, 10623 Berlin, Germany

## Abstract

**Background:**

Recent observational studies on volume-outcome associations in hepatobiliary surgery were not designed to account for the varying extent of hepatobiliary resections and the consequential risk of perioperative morbidity and mortality. Therefore, this study aimed to determine the risk-adjusted in-hospital mortality for minor and major hepatobiliary resections at the national level in Germany and to examine the effect of hospital volume on in-hospital mortality, and failure to rescue.

**Methods:**

All inpatient cases of hepatobiliary surgery (*n* = 31,114) in Germany from 2009 to 2015 were studied using national hospital discharge data. After ranking hospitals according to increasing hospital volumes, five volume categories were established based on all hepatobiliary resections. The association between hospital volume and in-hospital mortality following minor and major hepatobiliary resections was evaluated by multivariable regression methods.

**Results:**

Minor hepatobiliary resections were associated with an overall mortality rate of 3.9% and showed no significant volume-outcome associations. In contrast, overall mortality rate of major hepatobiliary resections was 10.3%. In this cohort, risk-adjusted in-hospital mortality following major resections varied widely across hospital volume categories, from 11.4% (95% CI 10.4–12.5) in very low volume hospitals to 7.4% (95% CI 6.6–8.2) in very high volume hospitals (risk-adjusted OR 0.59, 95% CI 0.41–0.54). Moreover, rates of failure to rescue decreased from 29.38% (95% CI 26.7–32.2) in very low volume hospitals to 21.38% (95% CI 19.2–23.8) in very high volume hospitals.

**Conclusions:**

In Germany, patients who are undergoing major hepatobiliary resections have improved outcomes, if they are admitted to higher volume hospitals. However, such associations are not evident following minor hepatobiliary resections. Following major hepatobiliary resections, 70–80% of the excess mortality in very low volume hospitals was estimated to be attributable to failure to rescue.

## Background

Numerous studies have identified hospital volume as significant independent variable of death in high-risk surgery [1–8]. As a result, several countries have implemented volume-based referral strategies. In some of these countries such initiatives have successfully led to an increasing regionalization of high-risk surgery that has contributed to declining mortality of several procedures, eg, esophagectomy and pancreatectomy in the US and Netherlands [4, 7].

In the past, volume-outcome analyses in hepatopancreatobiliary (HPB) surgery have usually focused on pancreatic surgery. Only a few studies have explicitly examined hospital volume effects in hepatobiliary surgery with contradictory results. On the one hand, US studies based on hospital discharge data from either State Inpatient Databases or the Nationwide Inpatient Sample found a significant association between higher hospital procedure volume and improved perioperative mortality [9–12]. On the other hand, Pal et al. found no significant differences in mortality between low and high volume hospitals in the UK using Hospital Episode Statistic data [13].

In hepatobiliary surgery, the extent of resection and the consequential risk of perioperative morbidity and mortality vary apparently. To date, observational outcome data on hepatobiliary surgery (excluding liver transplantation) that consider such variations are not available. Although, volume-outcome associations have been demonstrated for other high-risk surgical procedures at the national level in Germany [14–16], it is uncertain, whether centralization of care structures is beneficial for patients that undergo minor or major hepatobiliary surgery. As the promotion of volume-based referral (procedure volume as a quality indicator) may also have unintended side effects (eg, creating incentives for hospitals and surgeons to operate more often) [17], health care decision makers aiming to improve quality of care by centralization of services rely on objective evidence that higher volume is associated with better outcome [15]. This study, therefore, aimed to evaluate the association between hospital volume and in-hospital mortality following minor and major hepatobiliary resections using complete national hospital discharge data of Germany. In addition, the rate of complications and the risk of failure to rescue across hospital volume categories were analyzed.

## Methods

### Data

This study was based on individual inpatient data of the nationwide DRG (diagnosis-related groups) statistics that are provided by the Research Data Centers of the Federal Statistical Office and the state statistical offices [18]. Details on this nationwide database that is accessible via controlled remote data analysis have been described elsewhere. Every inpatient episode was assigned to the treating hospital based on an anonymized hospital identifier and included data from the years 2009 to 2015. Reporting of this study adheres to the REporting of studies Conducted using Observational Routinely-collected health Data (RECORD) statement [19].

This article does not contain any studies with human participants or animals performed by any of the authors. In accordance with the German guideline for conducting administrative data analyses ‚Good Practice in Secondary Data Analysis (GPS)’, no ethical approval was required for this study [20].

This study used administrative data provided by the Research Data Centre of the German Federal Statistical Office. In accordance with the terms of use regarding microdata provided by the Research Data Centers of the Federal Statistical Office and the Statistical Offices of the Federal States, no informed consent was required for this study https://www.forschungsdatenzentrum.de/en/terms-use.

### Patient population

**Fig. 1:**
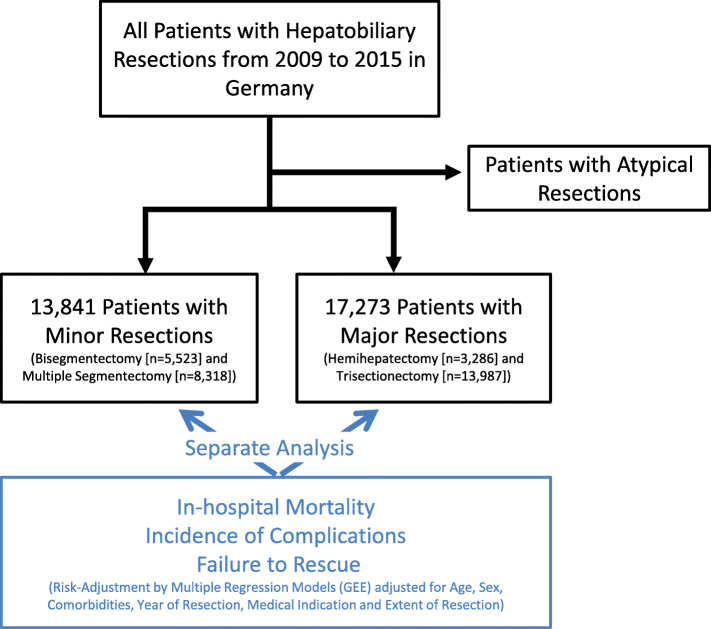
Study design

All inpatient episodes with the following types of liver resections from 2009 through 2015: Trisectionectomy (TS), hemihepatectomy (HH), multiple segmental resection (MSR) and bisegmentectomy (BS) were identified using appropriate procedure codes. Of these, TS and HH were defined as major resections, while MSR and BS were considered minor resections (Fig. 1). Surgeries such as atypical resections were excluded from this analysis, as the German operation procedure code (OPS) insufficiently separates larger atypical resections from small excision biopsies. All procedures were analyzed in hierarchical order within the same inpatient case in order to avoid double-counting a case as described previously [14]. Patients under the age of 20 and cases with procedure codes for post-mortem hepatectomy, liver transplantation, and liver graft resection were excluded from the analysis. Four groups of principal diagnoses (‘metastatic disease’, ‘primary malignant hepatobiliary neoplasm’ [including hepatocellular and cholangiocellular carcinoma], ‘benign neoplasm of the hepatobiliary system’ and ‘other medical indication’) were defined to stratify the analyses by medical indication. Inclusion and exclusion criteria as well as the definitions of medical indications and surgical procedures are listed in the Supplemental file 1.

### Hospital volume

Volume of hepatobiliary resections performed by a hospital was calculated for each year of observation. After ranking hospitals according to increasing hospital volumes, five volume categories were established by the selection of whole number cutoff points for annual volume. To allow a more precise breakdown of lower hospital volumes, these cut-offs were self-defined as follows: 1–10 resections = ‘very low’, 11–20 resections = ‘low’, 21–40 resections = ‘medium’, 41–100 resections = ‘high’ and > 100 = ‘very high’. The hospital ranking was based on all hepatobiliary resections and done for each year in order to allow adjustments of individual hospital ranks, if volume changed from 1 year to another.

### Outcome measure, risk adjustment and statistical analysis

Patient characteristics and all outcome measures were analyzed according to the volume categories and to the extent of hepatobiliary resection (minor and major resections). In-hospital mortality, defined as death before discharge, was studied as primary outcome measure. Observed and risk-adjusted mortality were stratified by volume categories. Risk-adjusted mortality for each volume category was calculated by multiplying the ratio of observed to expected mortality by the overall mortality. Expected mortality and risk-adjusted odds ratios (ORs) were calculated using logistic regression models that accounted for clustering and risk-adjustment as previously described in detail [14]. The exact definitions of covariates for risk adjustment (age, sex, year of treatment, comorbidities, medical indication, type of surgical procedure and extent of surgery) and associated odds ratios of in-hospital death are displayed in Supplemental file 2.

Failure to rescue (FTR) was studied as a secondary outcome, defined as in-hospital mortality among patients with complications or interventions required for complications, within each volume category. Three types of severe complications that are associated with a high risk of death were analyzed via secondary diagnosis codes (severe cardiovascular events [including myocardial infarction, stroke and pulmonary embolism], septic complications [including Peritonitis and Sepsis] and liver failure). Wherever possible, German operation procedure codes (OPS) of interventions required for complications were preferably used over ICD-10 codes of complications in order to increase reliability of the FTR analysis (eg, blood transfusion [6 or more units] instead of postoperative bleeding, mechanical ventilation [> 48 h] instead of acute respiratory insufficiency and hemodialysis [> 72 h] instead of acute renal failure). This approach is based on the fact that OPS codes are more relevant for reimbursement and thus better documented in administrative discharge data. In addition, the rate of percutaneous abdominal drainage was determined. Since a single patient case could involve more than one complication or intervention, a pooled rate of FTR was created representing all patient cases with at least one type of complication or intervention. Besides the observed FTR, the risk-adjusted FTR for these patients was calculated by means described above. The definitions of complications are displayed in Supplemental file 3.

The level of statistical significance was set to .05. The data analyses were conducted using SAS Version 9.3 (SAS Institute Inc., Cary, NC, USA).

## Results

### Patient characteristics

Between the years 2009 and 2015, a total of 31,114 inpatient cases with hepatobiliary resections were identified at the national level in Germany according to the above-mentioned including criteria. Of all procedures, 82.7% (*n* = 25,735) were performed for malignant indications including metastases. Benign disease and other diagnoses made up 7.9% (*n* = 2456) and 9.4% (*n* = 2923), respectively. More than half of all resections were TS and HH (10.6 and 45.0%, *n* = 17.273).

The annual absolute number of inpatient cases rose slightly from 4090 to 4432 over the 7-year time period (Table 1). The relative distribution of inpatient cases across hospital volume categories did not significantly vary between 2009 and 2015. Hospitals of the very low volume category (1–10 resections per year) treated 31.3% of all patients. The annual number of hospitals performing at least one hepatobiliary resection per year did not change over time. 81% of all hospitals did not perform more than 10 hepatobiliary resections per year.

**Table 1:** Characteristics of Patients Undergoing Hepatobiliary Surgery from 2009 to 2015, by Year

		2009	2010	2011	2012	2013	2014	2015
Total Number of Patients	N	4090	4283	4466	4654	4583	4606	4432
Minor Resection	N (%)	1833 (44.82)	1864 (43.52)	1915 (42.88)	2065 (44.37)	2045 (44.62)	2042 (44.33)	2077 (46.86)
Major Resection	N (%)	2257 (55.18)	2419 (56.48)	2551 (57.12)	2589 (55.63)	2538 (55.38)	2564 (55.67)	2355 (53.14)
Annual Number of Hospitals^a^	N	494	482	493	503	521	493	496
Demographics								
Age (Years)	Mean ± STD	62.86 ± 12.38	62.85 ± 12.68	62.83 ± 12.65	63.24 ± 12.75	63.21 ± 12.84	63.25 ± 1 2.68	63.33 ± 12.93
Age ≥ 65 Years	N (%)	2183 (53.37)	2239 (52.28)	2281 (51.07)	2472 (53.12)	2390 (52.15)	2396 (52.02)	2325 (52.46)
Female Sex	N (%)	1827 (44.67)	1849 (43.17)	1914 (42.86)	2081 (44.71)	1972 (43.03)	1975 (42.88)	1907 (43.03)
Underlying disease								
Metastatic Disease	N (%)	1916 (46.85)	2040 (47.63)	2023 (45.3)	2067 (44.41)	2022 (44.12)	2022 (43.9)	1919 (43.3)
Malignant Hepatobiliary Neoplasm	N (%)	1169 (28.58)	1317 (30.75)	1441 (32.27)	1505 (32.34)	1540 (33.6)	1568 (34.04)	1471 (33.19)
Benign Hepatobiliary Disease	N (%)	312 (7.63)	325 (7.59)	365 (8.17)	366 (7.86)	371 (8.1)	369 (8.01)	348 (7.85)
Other Medical Indication	N (%)	693 (16.94)	601 (14.03)	637 (14.26)	716 (15.38)	650 (14.18)	647 (14.05)	694 (15.66)
Type of Surgery								
Trisectionectomy	N (%)	394 (9.63)	472 (11.02)	444 (9.94)	486 (10.44)	509 (11.11)	498 (10.81)	483 (10.9)
Hemihepatectomy	N (%)	1863 (45.55)	1947 (45.46)	2107 (47.18)	2103 (45.19)	2029 (44.27)	2066 (44.85)	1872 (42.24)
Multiple Segmentectomy	N (%)	1163 (28.44)	1206 (28.16)	1219 (27.3)	1275 (27.4)	1140 (24.87)	1150 (24.97)	1165 (26.29)
Bisegmentectomy	N (%)	670 (16.38)	658 (15.36)	696 (15.58)	790 (16.97)	905 (19.75)	892 (19.37)	912 (20.58)
Extent of Surgery								
Resection of Arteries/Veins	N (%)	330 (8.07)	402 (9.39)	416 (9.31)	415 (8.92)	436 (9.51)	415 (9.01)	371 (8.37)
Biliodigestive Anastomosis	N (%)	117 (2.86)	136 (3.18)	211 (4.72)	195 (4.19)	173 (3.77)	184 (3.99)	180 (4.06)
Resection of Other Organ	N (%)	389 (9.51)	405 (9.46)	425 (9.52)	416 (8.94)	431 (9.4)	424 (9.21)	437 (9.86)

*STD* Standard Deviation

^a^That Performed at Least one Hepatobiliary Resection

On average 3 liver resections per year were performed by hospitals in the lowest volume category comprising between 392 and 428 institutions during the study period, while the small number of hospitals in the highest volume category (between 4 and 7 during the study period) performed 120 resections annually (Table 2 and Supplemental file 7). The rate of patients being transferred to another hospital did not differ between volume categories. Patients undergoing hepatobiliary resections in lower volume hospitals were older than those being treated in higher volume hospitals (Table 2). Notably, the proportion of patients aged 65 years or above was higher in low volume hospitals than in high volume hospitals (eg, very low: 58.4% vs. very high: 44.1%). The rate of metastatic disease as underlying disease was higher in lower volume hospitals, while benign diseases and malignant neoplasms were more frequently found in the higher volume categories. HH and TS were performed more often in high volume hospitals than in low volume hospitals (eg, very high: 67.67% vs. very low: 43.23%), as well as concomitant resections of visceral vessels (eg, very high: 7.62% vs. very low: 1.22%) and creations of hepatojejunal anastomosis (eg, very high: 18.11% vs. very low: 3.32%). BS and multiple segment resections were more frequent in very low volume hospitals compared to the other volume categories (eg, very high: 32.26% vs. very low: 56.77%). The frequency of coexisting conditions differed between volume categories regardless of whether minor or major resections were performed. A stratification of patient characteristics according to the extent of hepatobiliary resections (minor and major resections) is given in the Supplemental files 4 and 5.

**Table 2:** Characteristics of Patients Undergoing Hepatobiliary Resections from 2009 to 2015, According to Hospital Volume Categories

		Hospital Volume Categories				
		Very Low (1–10)	Low (11–20)	Medium (21–40)	High (41–100)	Very High (> 100)
Total Number of Patients	N	9734	4953	4648	6555	5224
Minor Resection	N	5526 (56.8)	2473 (49.9)	2002 (43.1)	2156 (32.9)	1684 (32.2)
Major Resection	N	4208 (43.2)	2480 (50.1)	2646 (56.9)	4399 (67.1)	3540 (67.8)
Annual Number of Hospitals^a^	Mean ± STD	403.3 ± 12.3	50.1 ± 5.2	24 ± 2.2	14.6 ± 1.6	5.4 ± 1.0
Annual Hospital Volume	Median (IQR)	3 (1–5)	13 (12–16)	27 (23–31)	63.5 (49–78)	119.5 (108–141)
Hospital to hospital transfer^b^						
Transfer-in	N (%)	176 (1.81)	109 (2.20)	142 (3.06)	418 (6.38)	322 (6.16)
Transfer-out	N (%)	448 (4.60)	205 (4.14)	187 (4.02)	318 (4.85)	206 (3.94)
Demographics						
Age (Years)	Mean ± STD	65.09 ± 0.12	64.29 ± 0.17	62.9 ± 0.19	61.46 ± 0.16	60.4 ± 10.18
Age ≥ 65 Years	N (%)	5683 (58.38)	2785 (56.23)	2419 (52.04)	3098 (47.26)	2301 (44.05)
Female Sex	N (%)	4248 (43.64)	2098 (42.36)	1947 (41.89)	2837 (43.28)	2395 (45.85)
Medical Indication						
Metastatic Disease	N (%)	4664 (47.91)	2439 (49.24)	2221 (47.78)	2764 (42.17)	1921 (36.77)
Malignant Hepatobiliary Neoplasm	N (%)	2553 (26.23)	1442 (29.11)	1444 (31.07)	2362 (36.03)	2210 (42.3)
Benign Hepatobiliary Disease	N (%)	711 (7.3)	344 (6.95)	366 (7.87)	583 (8.89)	452 (8.65)
Other Medical Indication	N (%)	1806 (18.55)	728 (14.7)	617 (13.27)	846 (12.91)	641 (12.27)
Comorbidities						
Chronic Heart Disease	N (%)	1152 (11.83)	573 (11.57)	466 (10.03)	569 (8.68)	506 (9.69)
Hypertension	N (%)	4654 (47.81)	2197 (44.36)	1980 (42.6)	2767 (42.21)	2244 (42.96)
Peripheral Vascular Disease	N (%)	167 (1.72)	71 (1.43)	57 (1.23)	90 (1.37)	74 (1.42)
Chronic Lung Disease	N (%)	737 (7.57)	299 (6.04)	301 (6.48)	347 (5.29)	327 (6.26)
Chronic Liver Disease	N (%)	947 (9.73)	514 (10.38)	541 (11.64)	843 (12.86)	798 (15.28)
Severe Kidney Disease	N (%)	853 (8.76)	383 (7.73)	355 (7.64)	414 (6.32)	258 (4.94)
Diabetes Mellitus	N (%)	1969 (20.23)	978 (19.75)	851 (18.31)	1151 (17.56)	1465 (28.04)
Obesity	N (%)	767 (7.88)	383 (7.73)	321 (6.91)	494 (7.54)	477 (9.13)
Coagulopathy	N (%)	109 (1.12)	67 (1.35)	69 (1.48)	79 (1.21)	27 (0.52)
Type of Surgery						
Trisectionectomy	N (%)	463 (4.76)	393 (7.93)	515 (11.08)	989 (15.09)	926 (17.73)
Hemihepatectomy	N (%)	3745 (38.47)	2087 (42.14)	2131 (45.85)	3410 (52.02)	2614 (50.04)
Multiple Segmentectomy	N (%)	3308 (33.98)	1517 (30.63)	1186 (25.52)	1272 (19.41)	1035 (19.81)
Bisegmentectomy	N (%)	2218 (22.79)	956 (19.3)	816 (17.56)	884 (13.49)	649 (12.42)
Extent of Surgery						
Resection of Arteries/Veins	N (%)	119 (1.22)	101 (2.04)	147 (3.16)	431 (6.58)	398 (7.62)
Biliodigestive Anastomosis	N (%)	323 (3.32)	299 (6.04)	375 (8.07)	842 (12.85)	946 (18.11)
Resection Other Organ	N (%)	781 (8.02)	494 (9.97)	457 (9.83)	626 (9.55)	569 (10.89)

^a^That Performed at Least one Hepatobiliary Resection

^b^Only Acute Care Hospitals. *STD* Standard Deviation, *IQR* Interquartile Range (25th to 75th percentile)

### In-hospital mortality

Overall in-hospital mortality was 7.5%. In the groups of minor and major resections, in-hospital mortality was on average 3.9 and 10.3%, respectively. TS were associated with the highest mortality, ranging from 8.6% in metastatic disease to 21.7% in case of malignant neoplasms (Table 3). Lowest mortality was found in patients undergoing BS for metastatic disease (1.0%).

**Table 3:** Number and In-hospital Mortality of Hepatobiliary Surgery Cases According to Types of Surgery and Medical Indication

		Metastatic Disease	Malignant Neoplasm	Benign Disease	Other Indication	Total
All Inpatients^a^	N	14,009	10,011	2456	4638	31,114
	Mortality (%)	(3.8)	(12.9)	(3.3)	(9.1)	(7.5)
Trisectionectomy	N	1211	1743	133	199	3286
	Mortality (%)	(8.6)	(21.7)	(8.3)	(17.6)	(16.1)
Hemihepatectomy	N	6509	4776	1019	1683	13,987
	Mortality (%)	(5.0)	(14.3)	(4.2)	(12.3)	(9.0)
Multiple Segmental Resection	N	4131	2253	584	1350	8318
	Mortality (%)	(2.1)	(7.1)	(2.4)	(5.8)	(4.1)
Bisegmentectomy	N	2158	1239	720	1406	5523
	Mortality (%)	(1.0)	(5.3)	(1.7)	(7.1)	(3.6)

^a^Undergoing Bisegmentectomy, Multiple Segmental Resection, Hemihepatectomy and Trisectionectomy

In-hospital mortality rates according to type of hepatobiliary resection and hospital volume categories are given in Supplemental file 8. Observed in-hospital mortality was inversely associated with hospital volume categories following major resections, ranging from 9.9% in the very high volume category to 11.6% in the very low volume category (Table 4). After risk adjustment the differences were clearly amplified and statistically significant (eg, very high: 7.4% [95% CI 6.6–8.2] vs. very low: 11.4% [95% CI 10.4–12.5]). In contrast, risk-adjusted rates of in-hospital mortality following minor resections did not significantly differ between low and high volume hospitals (eg, very high: 3.3% [95% CI 2.5–4.2] vs. very low: 4.5% [95% CI 4.0–5.1]) (Table 4). Further stratification according to the underlying diseases showed that the hospital volume effect found for major resections was primarily based on patients treated for malignant hepatobiliary neoplasms and metastatic disease (Table 5).

**Table 4:** Length of Stay, In-hospital Mortality and Failure to Rescue Following Minor and Major Hepatobiliary Resections, According to Hospital Volume Categories

		Hospital Volume Categories				
		Very Low (1–10)	Low (11–20)	Medium (21–40)	High (41–100)	Very High (> 100)
Minor Liver Surgery						
Length of Stay (Days)	Median (IQR)	14 (10–21)	13 (10–20)	12 (9–18)	13 (9–21)	12 (9–19)
In-hospital Mortality	n/N	247/5526	82/2473	60/2002	93/2156	57/1684
	Obs Rate %	4.5	3.3	3.0	4.3	3.4
	Adj Rate % (95% CI)	4.5 (4.0–5.1)	3.3 (2.6–4.1)	3.3 (2.5–4.2)	4.0 (3.2–4.8)	3.3 (2.5–4.2)
Incidence of Complications^a^	n/N	1137/5526	479/2473	391/2002	507/2156	408/1684
	Obs Rate %	20.58	19.37	19.53	23.52	24.23
Failure to Rescue^a^	n/N	208/1137	73/479	57/391	87/507	53/408
	Obs Rate %	18.29	15.24	14.58	17.16	12.99
	Adj Rate % (95% CI)	17.9 (15.6–20.5)	15.33 (12.0–19.3)	14.0 (10.6–18.1)	17.1 (13.7–21.1)	13.54 (10.1–17.7)
Major Liver Surgery						
Length of Stay (Days)	Median (IQR)	17 (12–28)	17 (12–29)	16 (12–28)	17 (12–28)	18 (12–30)
In-hospital Mortality	n/N	488/4208	269/2480	274/2646	406/4399	349/3540
	Obs Rate %	11.6	10.9	10.4	9.2	9.9
	Adj Rate % (95% CI)	11.4 (10.4–12.5)	10.1 (9.0–11.3)	9.19 (8.1–10.4)	8.0 (7.3–8.9)	7.4 (6.6–8.2)
Incidence of Complications^a^	n/N	1487/4208	893/2480	881/2646	1514/4399	1405/3540
	Obs Rate %	35.34	36.01	33.30	34.42	39.69
Failure to Rescue^a^	n/N	451/1487	259/893	268/881	394/1514	341/1405
	Obs Rate %	30.33	29.00	30.42	26.02	24.27
	Adj Rate % (95% CI)	29.38 (26.7–32.2)	27.13 (23.9–30.7)	28.05 (24.8–31.6)	24.0 (21.7–26.5)	21.38 (19.2–23.8)

Data are in n/N. Observed Rate (Obs Rate) in % and Adjusted Rate (Adj Rate) in % (95% CI). Covariates used for risk adjustment are displayed in Appendix Table 2

^a^Number of Treatment Cases with at Least one Complication (including Peritonitis, Sepsis, Liver Failure, Acute Myocardial Infarction, Stroke, Pulmonary Embolism, Hemodialysis [> 72 h], Prolonged Mechanical Ventilation [> 48 h], Blood Transfusions [≥6], Percutaneous Abdominal Drainage)

**Table 5:** In-hospital Mortality of Major Hepatobiliary Surgery According to Hospital Volume Categories, Stratified by Underlying Disease

		Hospital Volume Categories				
		Very Low (1–10)	Low (11–20)	Medium (21–40)	High (41–100)	Very High (> 100)
Major Liver Surgery						
All Indications	n/N	488/4208	269/2480	274/2646	406/4399	349/3540
	Obs Rate %	11.6	10.9	10.4	9.2	9.9
	Adj Rate % (95% CI)	11.4 (10.4–12.5)	10.1 (9.0–11.3)	9.19 (8.1–10.4)	8.0 (7.3–8.9)	7.4 (6.6–8.2)
Metastatic Disease	n/N	139/2149	74/1248	65/1221	91/1852	60/1250
	Obs Rate %	6.5	5.9	5.3	4.9	4.8
	Adj Rate % (95% CI)	6.5 (5.4–7.6)	5.4 (4.3–6.8)	5.02 (3.9–6.4)	4.83 (3.9–5.9)	4.67 (3.6–6.0)
Malignant Neoplasm	n/N	265/1286	148/813	159/982	250/1736	239/1702
	Obs Rate %	20.6	18.2	16.2	14.4	14.0
	Adj Rate % (95% CI)	18.8 (16.6–21.2)	15.8 (13.3–18.5)	13.34 (11.3–15.6)	11.86 (10.4–13.4)	10.59 (9.3–12.0)
Benign Disease	n/N	11/229	10/151	10/170	16/355	7/247
	Obs Rate %	4.8	6.6	5.9	4.5	2.8
	Adj Rate % (95% CI)	4.5 (2.3–8.1)	6.2 (3.0–11.3)	5.8 (2.8–10.6)	4.02 (2.3–6.5)	3.7 (1.5–7.6)
Other Medical	n/N	73/544	37/268	40/273	49/456	43/341
Indication^a^	Obs Rate %	13.4	13.8	14.7	10.8	12.6
	Adj Rate % (95% CI)	12.9 (10.1–16.2)	13.6 (9.6–18.7)	14.81 (10.6–20.2)	11.26 (8.3–14.9)	11.4 (8.2–15.3)

Data are in n/N. Observed Rates (Obs Rate) in % and Adjusted Rates (Adj Rate) in % (95% CI). Covariates used for risk adjustment are displayed in Appendix Table 2

^a^Including Liver Injury

**Fig. 2:**
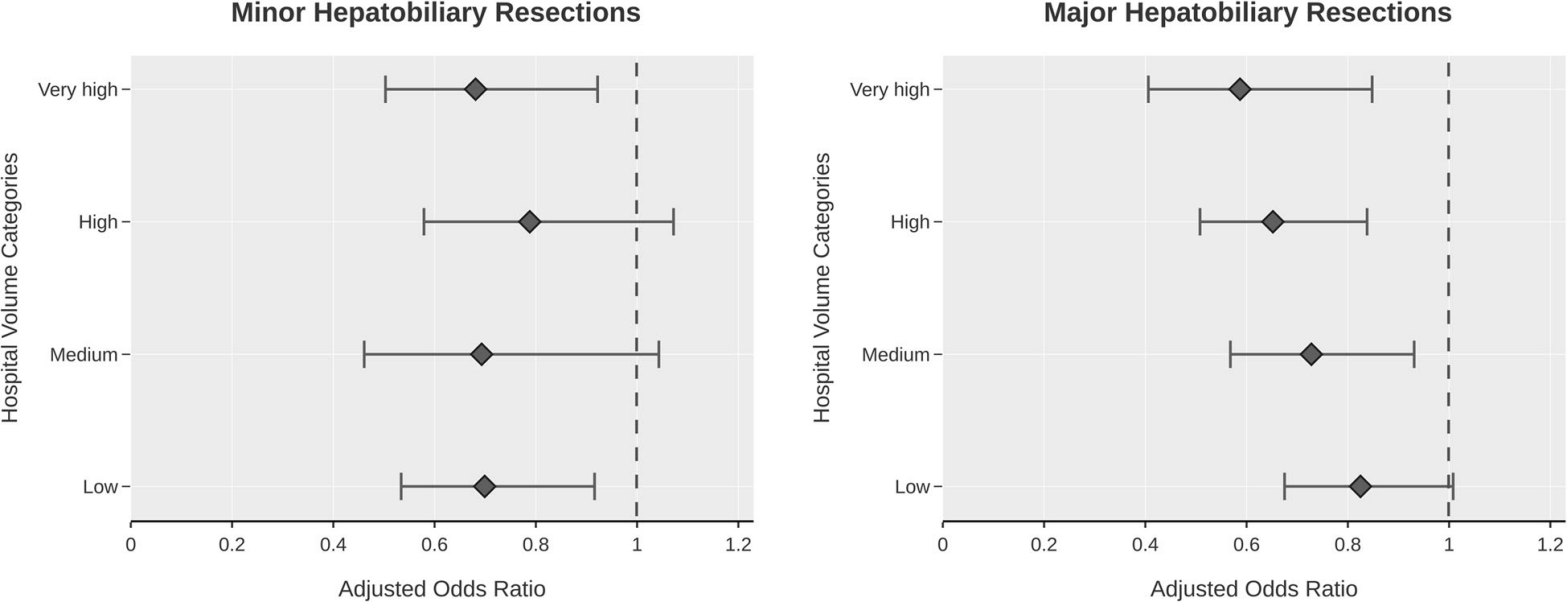
Risk-adjusted Odds Ratios of In-hospital Mortality of Minor and Major Hepatobiliary Resection Across Hospital Volume Categories. (Dashed Line Refers to the Very Low Hospital Volume Category [Reference])

After inclusion of hospital volume categories in the statistical model comprising relevant covariates, each higher volume category was associated with lower odds of in-hospital death when compared to the very low volume category in case of major resections. Such a distinct inverse relationship between hospital volume and odds of in-hospital death was not present in minor hepatobiliary surgery (Fig. 2). For major resections, the risk of dying was nearly halved (OR 0.59 [95% CI 0.4–0.9]) in hospitals with very high volumes as compared to hospitals with very low volumes. Still, the estimated reduction in mortality risk was 35% (OR 0.65 [95% CI 0.5–0.8]) and 27% (OR 0.73 [95% CI 0.6–0.9]) in hospitals with high and medium volumes, respectively. In case of minor resections, there was no significant reduction of the mortality risk except for the comparison of the very low category with the low volume (OR 0.7 [95% CI 0.5–0.9]) and very high volume category (OR 0.68 [95% CI 0.5–0.9]).

### Incidence of complications and failure to rescue

Incidence of complications in patients undergoing major hepatobiliary resections was higher than of those undergoing minor hepatobiliary surgeries. Overall, more than 29.3% of all patients had at least one complication. Rates of in-hospital mortality of patients with complications (failure to rescue) varied widely, ranging from 23% in patients with adverse vascular events (stroke or acute infarction or pulmonary embolism) to 90.7% in patients with a combination of adverse vascular events, septic disease and liver failure. In contrast, in-hospital mortality was only 2.6% in patients without complications.

The incidences of complications and observed mortality rates of patients with complications did not distinctively differ across hospital volume strata regardless of whether the patient received a minor or major resection (Supplemental file 6). However, observed mortality rates of patients with complications decreased with higher volumes. In case of major resections the risk-adjusted mortality of patients with at least one complication (pooled FTR) was significantly lower in very high volume hospitals (21.38 [95% CI 19.2–23.8) compared to low volume hospitals (27.13 [95% CI 23.9–30.7]) and very low volume hospitals (29.38 [95% CI 26.7–32.2], Table 4).

## Discussion

In the recent past, overall in-hospital mortality rates following hepatobiliary surgery reported by German single-institutional studies varied from 0 to 5.9% [21–25]. In contrast, the present population-based analysis revealed higher rates of in-hospital mortality in patients that underwent hepatobiliary surgery in Germany (eg, following minor resections = 3.9% and following major resections = 10.3%). In agreement with a previous nationwide analysis of pancreatic surgery outcomes, this discordancy may also based on publication bias [26].

In-hospital mortality rates reported by other international population-based studies are lower (3 to 3.7%) [9, 10, 27] compared to mortality rates of the current analysis. However, there are methodological differences, such as different databases and inclusion criteria, among these studies that do not allow adequate comparison, Of note, the present study included all adult patients undergoing hepatobiliary resections (≥2 segments) in German hospitals. In contrast, the studies mentioned above included patients that underwent non-anatomical wedge resections and HH (ICD-9 procedure codes 50.22 or 50.3) [9, 10, 27] or applied exclusion criteria such as non-solid neoplasms [9] or extrahepatic malignancies [27].

Analysis of volume-outcome relationships showed that hospital volume is not an independent significant predictor of death in patients that undergo minor hepatobiliary resections including BS and MSR. There was also no clear association between hospital volume and failure to rescue in this group. However, the current analysis revealed a remarkably significant reduction of in-hospital mortality following major hepatobiliary resections in higher volume hospitals in Germany. In the logistic regression models, major hepatobiliary surgery in hospitals of the very high volume category was associated with a 41% reduction in risk of in-hospital mortality compared with treatment in very low volume hospitals. As this hospital volume effect was primarily based on patients with malignant hepatobiliary neoplasms and metastatic disease, policy decisions that aim to improve outcomes through centralization should focus on these indications.

This study also revealed that the incidence of complications is not associated with the annual hospital volume. In case of major resections, however, the mortality rate of patients with complications (pooled FTR) was significantly lower in hospitals with higher volumes. Previous nationwide analyses of complex surgical procedures found that the management of patients with complications is an essential factor in explaining volume-outcome associations in high-risk general surgery [14–16]. In case of major hepatobiliary resections, 70–80% of the additional deaths of the very low volume category were estimated to be attributable to failure to rescue. These findings are also in line with various international analyses of other complex surgical procedures [27–30].

Post-hepatectomy liver failure (PHLF) is known to be a major cause of perioperative death. In the present analysis the incidence of clinically relevant PHLF (grade B and C) was 7.2% following major resections. In-hospital mortality in these patients was ranging from 52.8 to 90.7% depending on the existence of additional complications. Therefore, measures to improve perioperative outcomes need to focus on the prevention of PHLF. Of course, the incorporation of preoperative assessment of resectability and future liver remnant function into surgical decision-making is the essential key. In this regard, however, the question is: How much do economic incentives influence the willingness to push the limits in hepatobiliary surgery Germany?

The present analysis provides evidence that care structures of hepatobiliary surgery are currently not centralized in Germany. In accordance to previous findings in pancreatic, esophageal and gastric surgery [14, 16, 31], distribution of hospitals and variation of annual hospital volumes across the hospital volume categories showed a classical pattern of non-centralized care structures. In other words, more than 70% of all German hospitals performing hepatobiliary resections were found to have a very low volume (*n* ≤ 10). Since political efforts to centralize complex surgical procedures in Germany are currently ineffective [32] and minimum caseload requirements were not even defined for hepatobiliary surgery (in contrast to pancreatic and esophageal surgery), it is not surprising that there was no trend towards centralization over the observation period from 2009 to 2015 (Supplemental file 7). However, the presence of high in-hospital mortality and significant volume-outcome associations in major hepatobiliary surgery prompts the urgent need for strategies that aim to improve outcomes through centralization. One possible measure could be the introduction of minimal caseload requirements. If such a regulation were to be implemented, minimal caseload should be defined with a threshold of more than 20 major resections per year (banning hospitals of the very low and low volume categories from service).

Usually, tertiary referral hospitals claim to treat sicker patients than secondary care hospitals. Interestingly, patients treated in lower volume hospitals were older and tended to have more comorbidities than those treated in higher volume hospitals. These results correspond to previous findings in pancreatic surgery and may have multiple underlying causes (eg, younger patients willing to travel long distance to be treated in experienced facilities or possible incentives for low volume providers to operate patients with an elevated risk profile) that cannot be elucidated from the current data [14].

Another factor that is subject to considerable debate is whether higher volume hospitals perform more complex procedures compared to lower volume hospitals. Complexity of hepatobiliary surgery depends on several factors but may be estimated from the assessment of type of surgery (eg, HH or TS) and concomitant procedures (eg, vascular resections, creation of a hepatojejunal anastomosis and concomitant resection of other visceral organs). The fact that the rate of concomitant procedures is increasing across volume strata, while the rate of minor resections is decreasing, indicates an elevated level of surgical complexity of inpatient cases within the higher hospital volume categories. Nevertheless, in-hospital mortality rates of high volume hospitals (eg, metastatic disease: 4.67% and malignant hepatobiliary neoplasm: 10.59%) remain high. These unanticipated results prompt the need of outcomes research beyond volume-outcome relationships. Subsequently, other quality improvement strategies than centralization of care structures should be developed (eg, clinical peer review program).

The major strength of this study is the completeness of the data used. Limitations occur from the limited information available in administrative data as previously described [14]. Despite of a sophisticated risk adjustment model, unmeasured differences in comorbidity, degree of severity of diagnosis or appropriateness of surgical indication may partly explain the association between volume and outcome. This is, however, unlikely due to the fact that hospital volume and in-hospital mortality underlie a pattern of monotonic exposure-response relationship, which is considered as a strong argument for causal associations. Moreover, some definitions of ICD-10 codes are not sufficiently accurate to analyze postoperative complications (eg, postoperative bleeding). In such cases OPS codes of interventions required for complications were used instead (eg, transfusion of 6 units of blood). Another limitation of this study is the lack of data on surgeon volume, which has also been related with impaired perioperative outcomes in the past [9]. Finally, a possible misclassification of multi-campus hospitals as higher volume hospitals must be taken into account, resulting in a possible underestimation of the association between hospital volume and mortality [33].

## Conclusions

The current study indicates that the quality of care in major hepatobiliary surgery in Germany could be improved, if more patients were treated in hospitals with high annual operative volumes. Since the largest portion of the hospital volume-related differences of in-hospital mortality rest upon failure to rescue, additional efforts should focus on the improvement of complication management and the compliance with essential structural prerequisites.

## Supplementary information

**Additional file 1: Supplemental file 1.** Definition of Treatment Cases and Stratification Variables.

**Additional file 2: Supplemental file 2.** Definition of Indicators for Complications.

**Additional file 3: Supplemental file 3.** Definition of Covariates Used to Estimate Risk-Adjusted Mortality and Associated Odds Ratios of In-Hospital Mortality.

**Additional file 4: Supplemental file 4.** Characteristics of Patients Undergoing Minor Hepatobiliary Resections from 2009 to 2015, According to Hospital Volume Categories.

**Additional file 5: Supplemental file 5.** Characteristics of Patients Undergoing Major Hepatobiliary Resections from 2009 to 2015, According to Hospital Volume Categories.

**Additional file 6: Supplemental file 6.** Incidence and Death of Patients with Complications or Interventions Required for Complications According to Hospital Volume Categories.

**Additional file 7: Supplemental file 7.** Annual Distribution of Patients and Hospitals Across Hospital Volume Categories.

**Additional file 8: Supplemental file 8.** In-hospital Mortality According to Type of Hepatobiliary Resection and Hospital Volume Categories.

## Data Availability

The data that support the findings of this study are available from the Research Data Centers of the Federal Statistical Office and the state statistical offices (http://www.forschungsdatenzentrum.de/en/database/drg/index.asp) but restrictions apply to the availability of these data and so are not publicly available.

## Abbreviations

DRG: Diagnosis-related groups
ICD-10-GM: International Classification of Diseases, 10th Revision, German Modification
OPS: German operation procedure codes [Operationen- und Prozedurenschluessel] (based on the international classification of procedures in medicine, ICPM)
PE: Pulmonary embolism
RCT: Randomized controlled trial
AMI: Acute myocardial infarction
HPB: Hepatopancreatobiliary
PHLF: Post-hepatectomy liver failure
