# Supplementary material for: In-hospital mortality and failure to rescue following hepatobiliary surgery in Germany - a nationwide analysis
Source: BMC Surg. 2020 Jul 29;20:171. doi: 10.1186/s12893-020-00817-5 (PMC7388497; doi:10.1186/s12893-020-00817-5)
Supplement: Supplementary file 1 — Additional file 1: Supplemental file 1. Definition of Treatment Cases and Stratification Variables. [file 12893_2020_817_MOESM1_ESM.docx]

| **Supplemental File 1. Definition of Treatment Cases and Stratification Variables.** | | |
| --- | --- | --- |
|  |  |  |
|  | **Inclusion** | **Exclusion** |
|  |  |  |
| Patient Population (All Major Liver Resections) | OPS 55026 55023 55021 55022 55025 55024 | OPS 5503 5504 Age <20 |
|  |  |  |
| Type of Surgery* |  |  |
| Trisectionectomy | OPS 55026 55023 |  |
| Hemihepatectomy | OPS 55021 55022 | OPS 55026 55023 |
| Multiple Segmental Resection | OPS 55025 | OPS 55026 55023 55021 55022 |
| Bisegmentectomy | OPS 55024 | OPS 55026 55023 55021 55022 55025 |
|  |  |  |
| Extended Surgery |  |  |
| Biliodigestive Anastomosis | OPS 5512 |  |
| Resection of Visceral Arteries/Venes | OPS 53826 53829 53836 53839 53956 53959 53966 53976 53979 |  |
| Concomitant Resection of Visceral Organs Other than Liver | OPS 5072 532 5347 5413 5436 5437 54540 54541 54542 54543 54544 54545 54546 5454x 5454y 5455 5456 5524 5525 5554 |  |
|  |  |  |
| Medical Indication (Principal Diagnosis) |  |  |
| Metastatic Disease | ICD C77 C78 C79 |  |
| Malignant Hepatobiliary Neoplasm | ICD C22 C23 C24 |  |
| Benign Hepatobiliary Disease | ICD B670 B673 B674 B675 B676 B677 B678 B679 D134 D135 D376 D3778 D015 K768 Q446 K750 |  |
| Other Medical Indication** | All other Principal Diagnoses |  |
|  |  |  |
| ICD-10-GM, International Statistical Classification of Diseases and Related Health Problems, 10^th^ revision, German modification; OPS, German Procedure Classification;  *If two or more procedure codes were present the higher hierarchy level was given to the most radical procedure. **Including Traumatic Liver Injuries. | | |
