# Supplementary material for: In-hospital mortality and failure to rescue following hepatobiliary surgery in Germany - a nationwide analysis
Source: BMC Surg. 2020 Jul 29;20:171. doi: 10.1186/s12893-020-00817-5 (PMC7388497; doi:10.1186/s12893-020-00817-5)
Supplement: Supplementary file 2 — Additional file 2: Supplemental file 2. Definition of Indicators for Complications. [file 12893_2020_817_MOESM2_ESM.docx]

| **Supplemental File 2. Definition of Covariates Used to Estimate Risk-Adjusted Mortality and Associated Odds Ratios of In-Hospital Death.** | | | | | |
| --- | --- | --- | --- | --- | --- |
|  | |  | **Odds Ratios of In-Hospital Death** | | |
| **Covariate** | | **Definition** | **Minor Resections** | **Major Resections** |  |
| Calendar Year of Treatment | |  |  |  |  |
| 2009 | |  | 1.1 (0.9 - 1.3) | 1.4 (0.9 - 1.9) |  |
| 2010 | |  | 1.1 (0.9 - 1.4) | 1.2 (0.9 - 1.8) |  |
| 2011 | |  | 1.1 (0.9 - 1.3) | 1.6 (1.1 - 2.2) |  |
| 2012 | |  | 1.2 (1.0 - 1.4) | 1.5 (1.1 - 2.2) |  |
| 2013 | |  | 1.0 (0.8 - 1.2) | 1.2 (0.9 - 1.7) |  |
| 2014 | |  | 1.2 (1.0 - 1.5) | 1.3 (0.9 - 1.9) |  |
| 2015 | |  | Reference | Reference |  |
|  | |  |  |  |  |
| Age^1^ | |  | 1.05 (1.04 - 1.06) | 1.05 (1.04 - 1.06) |  |
|  | |  |  |  |  |
| Sex^2^ | |  | 1.4 (1.2 - 1.5) | 1.1 (0.9 - 1.4) |  |
|  | |  |  |  |  |
| Medical Indication (Principal Diagnosis) | |  |  |  |  |
| Metastatic Disease | | See Appendix Table 1 | Reference | Reference |  |
| Malignant Neoplasm of Liver | | See Appendix Table 1 | 1.9 (1.6 - 2.2) | 2.3 (1.7 - 3.0) |  |
| Benign Disease of Liver | | See Appendix Table 1 | 1.2 (0.9 - 1.6) | 1.8 (1.1 - 2.7) |  |
| Other Medical Indication | | See Appendix Table 1 | 2.4 (2.0 - 2.9) | 3.4 (2.6 - 4.4) |  |
|  | |  |  |  |  |
| Coexisting Condition (Secondary Diagnosis)^3^ | |  |  |  |  |
| Chronic Heart Disease | | ICD I25 I420 I426 I427 I428 I429 I340 I342 I350 I351 I352 I050 I051 I052 I060 I061 I062 Q231 Q232 Q233 Z450 Z950 | 1.4 (1.2 - 1.6) | 1.9 (1.5 - 2.4) |  |
| Hypertension | | ICD I10 I15 | 0.8 (0.7 - 0.9) | 0.7 (0.6 - 0.8) |  |
| Peripheral Vascular Disease | | ICD I702 | 1.3 (0.9 - 2.0) | 1.7 (1.0 - 2.8) |  |
| Chronic Pulmonary Disease | | ICD J41 J42 J44 J45 J47 | 1.2 (0.9 - 1.4) | 1.3 (0.9 - 1.7) |  |
| Chronic Liver Disease | | ICD B18 I864 I982 K70 K73 K74 K760 K761 K765 K766 K767 Q446 Q447 | 2.0 (1.7 - 2.4) | 2.4 (1.9 - 3.0) |  |
| Severe Renal Disease | | ICD I120 N03 N04 N05 N07 N08 N11 N12 N14 N15 N16 N18 Z992 | 1.5 (1.2 - 1.7) | 1.7 (1.3 - 2.2) |  |
| Diabetes Mellitus | | ICD E10 E11 E12 E13 E14 | 1.3 (1.2 - 1.5) | 0.9 (0.8 - 1.2) |  |
| Obesity | | ICD E66 | 1.0 (0.9 - 1.2) | 0.7 (0.5 - 1.0) |  |
| Coagulopathy | | ICD D66 D67 D680 D681 D682 D685 D691 D693 D694 | 2.4 (1.6 - 3.7) | 4.2 (2.2 - 8.2) |  |
|  |  | |  |  |  |
| Extent of Surgery^4^ |  | |  |  |  |
| Biliodigestive Anastomosis | | See Appendix Table 1 | 2.5 (2.1 - 3.0) | 3.6 (2.6 - 5.1) |  |
| Resection of Visceral Arteries/Veins | | See Appendix Table 1 | 2.3 (1.9 - 2.7) | 3.4 (1.8 - 6.5) |  |
| Concomitant Resection of Visceral Organs | | See Appendix Table 1 | 2.1 (1.8 - 2.5) | 3.1 (2.4 - 3.9) |  |
|  | |  |  |  |  |
| Area under the Curve (c-statistic) | |  | 0.779 | 0.775 |  |
|  | |  |  |  |  |
| ICD-10-GM, International Statistical Classification of Diseases and Related Health Problems, 10^th^ revision, German modification. ^1^5-year Age Groups  References: ^2^Female Sex, ^3^No Coexisting Condition, ^4^No Extent of Surgery | | | | | |
