# Supplementary material for: In-hospital mortality and failure to rescue following hepatobiliary surgery in Germany - a nationwide analysis
Source: BMC Surg. 2020 Jul 29;20:171. doi: 10.1186/s12893-020-00817-5 (PMC7388497; doi:10.1186/s12893-020-00817-5)
Supplement: Supplementary file 3 — Additional file 3: Supplemental file 3. Definition of Covariates Used to Estimate Risk-Adjusted Mortality and Associated Odds Ratios of In-Hospital Mortality. [file 12893_2020_817_MOESM3_ESM.docx]

| **Supplemental File 3. Definition of Indicators for Complications.** | |
| --- | --- |
| **Indicator** | **Definition** |
| Complications (Secondary Diagnosis) |  |
| Stroke or AMI or PE | ICD I21 I26 I60 I61 I63 I64 |
| Peritonitis or Septicaemia | ICD K65 A40 A41 R572 R65 |
| Liver Failure | K720 K729 |
|  |  |
| Interventions Required for Complications |  |
| Blood Transfusions (≥6) | OPS 8800c1-8800cr 880070-88007e 88007g 88007h |
| Mechanical Ventilation (> 48h) | Value from Respective Data Field >48 |
| Hemodialysis (>72h) | OPS 885372-7c 885382-8c 885462-6c 885472-7c 885572-7c 885582-8c |
| Percutaneous Abdominal Drainage | OPS 8146 8148 8153 8154 |
|  |  |
| ICD-10-GM, International Statistical Classification of Diseases and Related Health Problems, 10^th^ revision, German modification; OPS, German Procedure Codes; | |
