# Supplementary material for: In-hospital mortality and failure to rescue following hepatobiliary surgery in Germany - a nationwide analysis
Source: BMC Surg. 2020 Jul 29;20:171. doi: 10.1186/s12893-020-00817-5 (PMC7388497; doi:10.1186/s12893-020-00817-5)
Supplement: Supplementary file 4 — Additional file 4: Supplemental file 4. Characteristics of Patients Undergoing Minor Hepatobiliary Resections from 2009 to 2015, According to Hospital Volume Categories. [file 12893_2020_817_MOESM4_ESM.docx]

| **Supplemental File 4. Characteristics of Patients Undergoing Minor Hepatobiliary Resections from 2009 to 2015, According to Hospital Volume Categories.** | | | | | | |  |
| --- | --- | --- | --- | --- | --- | --- | --- |
|  |  | **Hospital Volume Quintiles** | | | | |  |
|  |  | **Very Low**  (1-10) | **Low**  (11-20) | **Medium**  (21-40) | **High**  (41-100) | **Very High** (>100) |  |
|  |  |  |  |  |  |  |  |
| Number of Patients | N | 5526 | 2473 | 2002 | 2156 | 1684 |  |
|  |  |  |  |  |  |  |  |
| Hospital to hospital transfer^+^ |  |  |  |  |  |  |  |
| Transfer-in | N (%) | 104 (1.88) | 45 (1.82) | 42 (2.10) | 114 (5.29) | 61 (3.62) |  |
| Transfer-out | N (%) | 212 (3.84) | 85 (3.44) | 67 (3.35) | 96 (4.45) | 62 (3.68) |  |
|  |  |  |  |  |  |  |  |
| Demographics |  |  |  |  |  |  |  |
| Age (Years) | Mean ± STD | 65.2 ± 0.2 | 64.5 ± 0.3 | 62.6 ± 0.3 | 61.2 ± 0.3 | 59.9 ± 0.3 | |
| Age ≥65 Years | N (%) | 3228 (58.4) | 1416 (57.3) | 1034 (51.7) | 1006 (46.7) | 739 (43.9) | |
| Female Sex | N (%) | 2478 (44.8) | 1077 (43.6) | 848 (42.4) | 939 (43.6) | 810 (48.1) | |
|  |  |  |  |  |  |  | |
| Medical Indication |  |  |  |  |  |  | |
| Metastatic Disease | N (%) | 2515 (45.51) | 1191 (48.16) | 1000 (49.95) | 912 (42.30) | 671 (39.85) | |
| Malignant Neoplasm | N (%) | 1267 (22.93) | 629 (25.43) | 462 (23.08) | 626 (29.04) | 508 (30.17) | |
| Benign Disease | N (%) | 482 (8.72) | 193 (7.80) | 196 (9.79) | 228 (10.58) | 205 (12.17) | |
| Other Medical Indication | N (%) | 1262 (22.84) | 460 (18.60) | 344 (17.18) | 390 (18.09) | 300 (17.81) | |
|  |  |  |  |  |  |  |  |
| Comorbidities |  |  |  |  |  |  |  |
| Chronic Heart Disease | N (%) | 671 (12.14) | 306 (12.37) | 192 (9.59) | 177 (8.21) | 151 (8.97) |  |
| Hypertension | N (%) | 2663 (48.19) | 1103 (44.6) | 833 (41.61) | 881 (40.86) | 708 (42.04) |  |
| Peripheral Vascular Disease | N (%) | 94 (1.7) | 42 (1.7) | 15 (0.75) | 32 (1.48) | 21 (1.25) |  |
| Chronic Lung Disease | N (%) | 411 (7.44) | 159 (6.43) | 143 (7.14) | 111 (5.15) | 95 (5.64) |  |
| Chronic Liver Disease | N (%) | 562 (10.17) | 290 (11.73) | 249 (12.44) | 292 (13.54) | 294 (17.46) |  |
| Severe Kidney Disease | N (%) | 471 (8.52) | 200 (8.09) | 139 (6.94) | 139 (6.45) | 91 (5.4) |  |
| Diabetes Mellitus | N (%) | 1141 (20.65) | 530 (21.43) | 389 (19.43) | 391 (18.14) | 399 (23.69) |  |
| Obesity | N (%) | 458 (8.29) | 203 (8.21) | 141 (7.04) | 157 (7.28) | 189 (11.22) |  |
| Coagulopathy | N (%) | 44 (0.8) | 21 (0.85) | 15 (0.75) | 15 (0.7) | 9 (0.53) |  |
|  |  |  |  |  |  |  |  |
| Type of Surgery |  |  |  |  |  |  |  |
| Multiple Segmentectomy | N (%) | 3308 (59.9) | 1517 (61.3) | 1186 (59.2) | 1272 (59.0) | 1035 (61.5) |  |
| Bisegmentectomy | N (%) | 2218 (40.1) | 956 (38.7) | 816 (40.8) | 884 (41.0) | 649 (38.5) |  |
|  |  |  |  |  |  |  |  |
| Extent of Surgery |  |  |  |  |  |  |  |
| Resection of Arteries/Veins | N (%) | 61 (1.1) | 40 (1.62) | 38 (1.9) | 130 (6.03) | 91 (5.4) |  |
| Biliodigestive Anastomosis | N (%) | 28 (0.51) | 13 (0.53) | 23 (1.15) | 90 (4.17) | 45 (2.67) |  |
| Resection Other Organ | N (%) | 435 (7.87) | 231 (9.34) | 167 (8.34) | 216 (10.02) | 188 (11.16) |  |
|  |  |  |  |  |  |  |  |
| ^*^That Performed at Least one Hepatobiliary Resection.  ^+^Only Acute Care Hospitals. STD – Standard Deviation | | | | | | |  |
