# Supplementary material for: In-hospital mortality and failure to rescue following hepatobiliary surgery in Germany - a nationwide analysis
Source: BMC Surg. 2020 Jul 29;20:171. doi: 10.1186/s12893-020-00817-5 (PMC7388497; doi:10.1186/s12893-020-00817-5)
Supplement: Supplementary file 5 — Additional file 5: Supplemental file 5. Characteristics of Patients Undergoing Major Hepatobiliary Resections from 2009 to 2015, According to Hospital Volume Categories. [file 12893_2020_817_MOESM5_ESM.docx]

| **Supplemental File 5. Characteristics of Patients Undergoing Major Hepatobiliary Resections from 2009 to 2015, According to Hospital Volume Categories.** | | | | | | |  |
| --- | --- | --- | --- | --- | --- | --- | --- |
|  |  | **Hospital Volume Quintiles** | | | | |  |
|  |  | **Very Low**  (1-10) | **Low**  (11-20) | **Medium**  (21-40) | **High**  (41-100) | **Very High** (>100) |  |
|  |  |  |  |  |  |  |  |
| Total Number of Patients | N | 4208 | 2480 | 2646 | 4399 | 3540 | |
|  |  |  |  |  |  |  | |
| Hospital to hospital transfer^+^ |  |  |  |  |  |  | |
| Transfer-in | N (%) | 72 (1.71) | 64 (2.58) | 100 (3.78) | 304 (6.91) | 261 (7.37) | |
| Transfer-out | N (%) | 236 (5.61) | 120 (4.84) | 120 (4.54) | 222 (5.05) | 144 (4.07) | |
|  |  |  |  |  |  |  | |
| Demographics |  |  |  |  |  |  | |
| Age (Years) | Mean ±STD | 65.0 ± 0.2 | 64.13 ± 0.2 | 63.1 ± 0.2 | 61.6 ± 0.2 | 60.6 ± 0.2 | |
| Age ≥65 Years | N (%) | 2455 (58.3) | 1369 (55.2) | 1385 (52.3) | 2092 (47.6) | 1562 (44.1) | |
| Female Sex | N (%) | 1770 (42.1) | 1021 (41.2) | 1099 (41.5) | 1898 (43.2) | 1585 (44.8) | |
|  |  |  |  |  |  |  | |
| Medical Indication |  |  |  |  |  |  | |
| Metastatic Disease | N (%) | 2149 (51.07) | 1248 (50.32) | 1221 (46.15) | 1852 (42.10) | 1250 (35.31) | |
| Malignant Neoplasm | N (%) | 1286 (30.56) | 813 (32.78) | 982 (37.11) | 1736 (39.46) | 1702 (48.08) | |
| Benign Disease | N (%) | 229 (5.44) | 151 (6.09) | 170 (6.42) | 355 (8.07) | 247 (6.98) | |
| Other Medical Indication | N (%) | 544 (12.93) | 268 (10.81) | 273 (10.32) | 456 (10.37) | 341(9.63) | |
|  |  |  |  |  |  |  | |
| Comorbidities |  |  |  |  |  |  | |
| Chronic Heart Disease | N (%) | 481 (11.43) | 267 (10.77) | 274 (10.36) | 392 (8.91) | 355 (10.03) | |
| Hypertension | N (%) | 1991 (47.3) | 1094 (44.11) | 1147 (43.35) | 1886 (42.87) | 1536 (43.39) | |
| Peripheral Vascular Disease | N (%) | 73 (1.73) | 29 (1.17) | 42 (1.59) | 58 (1.32) | 53 (1.5) | |
| Chronic Lung Disease | N (%) | 326 (7.75) | 140 (5.65) | 158 (5.97) | 236 (5.36) | 232 (6.55) | |
| Chronic Liver Disease | N (%) | 385 (9.15) | 224 (9.03) | 292 (11.04) | 551 (12.53) | 504 (14.24) | |
| Severe Kidney Disease | N (%) | 382 (9.08) | 183 (7.38) | 216 (8.16) | 275 (6.25) | 167 (4.72) | |
| Diabetes Mellitus | N (%) | 828 (19.7) | 448 (18.06) | 462 (17.46) | 760 (17.28) | 1066 (30.11) | |
| Obesity | N (%) | 309 (7.34) | 180 (7.26) | 180 (6.8) | 337 (7.66) | 288 (8.14) | |
| Coagulopathy | N (%) | 65 (1.54) | 46 (1.85) | 54 (2.04) | 64 (1.45) | 18 (0.51) | |
|  |  |  |  |  |  |  | |
| Type of Surgery |  |  |  |  |  |  | |
| Trisectionectomy | N (%) | 463 (11.0) | 393 (15.8) | 515 (19.5) | 989 (22.5) | 926 (26.2) | |
| Hemihepatectomy | N (%) | 3745 (89.0) | 2087 (84.2) | 2131 (80.5) | 3410 (77.5) | 2614 (73.8) | |
|  |  |  |  |  |  |  | |
| Extent of Surgery |  |  |  |  |  |  | |
| Resection of Arteries/Veins | N (%) | 91 (2.16) | 88 (3.55) | 124 (4.69) | 341 (7.75) | 353 (9.97) | |
| Biliodigestive Anastomosis | N (%) | 262 (6.23) | 259 (10.44) | 337 (12.74) | 712 (16.19) | 855 (24.15) | |
| Resection Other Organ | N (%) | 346 (8.22) | 263 (10.6) | 290 (10.96) | 410 (9.32) | 381 (10.76) | |
|  |  |  |  |  |  |  |  |
| ^*^That Performed at Least one Hepatobiliary Resection.  ^+^Only Acute Care Hospitals. STD – Standard Deviation | | | | | | |  |
