# Supplementary material for: In-hospital mortality and failure to rescue following hepatobiliary surgery in Germany - a nationwide analysis
Source: BMC Surg. 2020 Jul 29;20:171. doi: 10.1186/s12893-020-00817-5 (PMC7388497; doi:10.1186/s12893-020-00817-5)
Supplement: Supplementary file 6 — Additional file 6: Supplemental file 6. Incidence and Death of Patients with Complications or Interventions Required for Complications According to Hospital Volume Categories. [file 12893_2020_817_MOESM6_ESM.docx]

| **Supplemental File 6. Incidence and Death of Patients with Complications or Interventions Required for Complications According to Hospital Volume Categories.** | | | | | | |
| --- | --- | --- | --- | --- | --- | --- |
|  |  | **Hospital Volume Categories** | | | | |
|  |  | Very Low (1-10) | Low (11-20) | Medium (21-40) | High (41-100) | Very High (>100) |
| Minor Liver Surgery |  |  |  |  |  |  |
| Stroke or AMI or PE | n/N | 21/94 | 9/33 | 4/41 | 13/57 | 8/30 |
|  | Obs Rate % | 22.34 (13.9 - 30.8) | 27.27 (12.1 - 42.5) | 9.76% (0.67 - 18.84) | 22.81 (11.9 - 33.7) | 26.67 (10.8 - 42.5) |
| Peritonitis or Septicemia | n/N | 123/470 | 40/188 | 38/144 | 52/187 | 38/176 |
|  | Obs Rate % | 26.17 (22.2 - 30.1) | 21.28% (15.4 - 27.1) | 26.39 (19.2 - 33.6) | 27.81 (21.4 - 34.2) | 21.59 (15.5 - 27.7) |
| Liver Failure | n/N | 53/85 | 18/29 | 18/25 | 33/51 | 17/34 |
|  | Obs Rate % | 62.35 (52.1 - 72.7) | 62.07 (44.4 - 79.7) | 72 (54.4 - 89.6) | 64.71 (51.6 - 77.8) | 50.0 (33.2 - 66.8) |
| Hemodialysis (>72h) | n/N | 34/53 | 14/22 | 12/15 | 17/27 | 14/22 |
|  | Obs Rate % | 64.15 (51.2 - 77.1) | 63.64 (43.5 - 83.7) | 80 (59.8 - 100.2) | 62.96 (44.8 - 81.2) | 63.64 (43.5 - 83.7) |
| Mechanical Ventilation (>48h) | n/N | 133/342 | 50/135 | 34/87 | 59/121 | 37/90 |
|  | Obs Rate % | 38.89 (33.7 - 44.1) | 37.04 (28.9 - 45.2) | 39.08 (28.8 - 49.3) | 48.76 (39.9 - 57.7) | 41.11 (31.0 - 51.3) |
| Blood Transfusions (≥6) | n/N | 138/544 | 56/233 | 36/168 | 62/201 | 45/183 |
|  | Obs Rate % | 25.37 (21.7 - 29.0) | 24.03 (18.6 - 29.5) | 21.43 (15.2 - 27.6) | 30.85 (24.5 - 37.2) | 24.59 (18.4 - 30.8) |
| Percutaneous Drainage | n/N | 17/316 | 11/158 | 12/159 | 25/237 | 17/186 |
|  | Obs Rate % | 5.38 (3.0 - 7.9) | 6.96 (3.0 - 10.9) | 7.55 (3.4 - 11.7) | 10.55 (6.6 - 14.5) | 9.14 (5.0 - 13.3) |
|  |  |  |  |  |  |  |
| Major Liver Surgery |  |  |  |  |  |  |
| Stroke or AMI or PE | n/N | 54/141 | 18/68 | 41/90 | 61/171 | 43/107 |
|  | Obs Rate % | 38.3 (30.3 - 46.3) | 26.47 (16.0 - 37.0) | 45.56 (35.3 - 55.8) | 35.67 (28.5 - 42.9) | 40.19 (30.9 - 49.5) |
| Peritonitis or Septicemia | n/N | 240/609 | 145/361 | 176/406 | 261/639 | 245/711 |
|  | Obs Rate % | 39.41 (35.5 - 43.3) | 40.17 (35.1 - 45.2) | 43.35 (38.5 - 48.2) | 40.85 (37.0 - 44.7) | 34.46 (31.0 - 38.0) |
| Liver Failure | n/N | 191/294 | 134/189 | 146/194 | 203/324 | 177/241 |
|  | Obs Rate % | 64.97 (59.5 - 70.4) | 70.9 (64.4 - 77.4) | 75.26 (69.2 - 81.3) | 62.65 (57.4 - 67.9) | 73.44 (67.9 - 79.0) |
| Hemodialysis (>72h) | n/N | 81/102 | 75/93 | 77/88 | 114/140 | 126/150 |
|  | Obs Rate % | 79.4 (71.6 - 87.3) | 80.65 (72.6 - 88.7) | 87.5 (80.6 - 94.4) | 81.43 (75.0 - 87.9) | 84 (78.1 - 89.9) |
| Mechanical Ventilation (>48h) | n/N | 305/533 | 173/308 | 193/315 | 289/499 | 257/400 |
|  | Obs Rate % | 57.22 (53.0 - 61.4) | 56.17 (50.6 - 61.7) | 61.27 (55.9 - 66.7) | 57.92 (53.6 - 62.3) | 64.25 (59.6 - 69.0) |
| Blood Transfusions (≥6) | n/N | 323/808 | 172/486 | 203/489 | 316/725 | 287/751 |
|  | Obs Rate % | 39.98 (36.6 - 43.4) | 35.39 (31.1 - 39.6) | 41.5 (37.2 - 45.9) | 43.59 (40.0 - 47.2) | 38.22 (34.7 - 41.7) |
| Percutaneous Drainage | n/N | 52/410 | 47/287 | 55/300 | 93/621 | 112/647 |
|  | Obs Rate % | 12.68 (9.5 - 16.0) | 16.38 (12.1 - 20.7) | 18.3 (14.0 - 22.7) | 14.98 (12.2 - 17.8) | 17.31 (14.4 - 20.2) |
|  |  |  |  |  |  |  |
| Data are in n/N. Observed Rate (Obs Rate) in %. | | | | | | |
