# Supplementary material for: In-hospital mortality and failure to rescue following hepatobiliary surgery in Germany - a nationwide analysis
Source: BMC Surg. 2020 Jul 29;20:171. doi: 10.1186/s12893-020-00817-5 (PMC7388497; doi:10.1186/s12893-020-00817-5)
Supplement: Supplementary file 7 — Additional file 7: Supplemental file 7. Annual Distribution of Patients and Hospitals Across Hospital Volume Categories. [file 12893_2020_817_MOESM7_ESM.docx]

| **Supplemental File 7. Annual Distribution of Patients and Hospitals Across Hospital Volume Categories.** | | | | | | | | | | |  |
| --- | --- | --- | --- | --- | --- | --- | --- | --- | --- | --- | --- |
|  | **Hospital Volume Categories** | | | | | | | | | |  |
|  | **Very Low (1-10)** | | **Low (11-20)** | | **Medium (21-40)** | | **High (41-100)** | | **Very High (>100)** | | **Total** |
|  | n | (%) | n | (%) | n | (%) | n | (%) | n | (%) | n |
| Nationwide Patient Distribution per Year | | |  |  |  |  |  |  |  |  |  |
| 2009 | 1361 | (33.3) | 649 | (15.9) | 636 | (15.6) | 740 | (18.1) | 704 | (17.2) | 4090 |
| 2010 | 1294 | (30.2) | 685 | (16.0) | 613 | (14.3) | 1003 | (23.4) | 688 | (16.1) | 4283 |
| 2011 | 1333 | (29.8) | 678 | (15.2) | 719 | (16.1) | 918 | (20.6) | 818 | (18.3) | 4466 |
| 2012 | 1359 | (29.2) | 823 | (17.7) | 651 | (14.0) | 994 | (21.4) | 827 | (17.8) | 4654 |
| 2013 | 1531 | (33.4) | 705 | (15.4) | 621 | (13.6) | 1014 | (22.1) | 712 | (15.5) | 4583 |
| 2014 | 1422 | (30.9) | 730 | (15.8) | 666 | (14.5) | 858 | (18.6) | 930 | (20.2) | 4606 |
| 2015 | 1434 | (32.4) | 683 | (15.4) | 742 | (16.7) | 1028 | (23.2) | 545 | (12.3) | 4432 |
|  |  |  |  |  |  |  |  |  |  |  |  |
| Nationwide Hospital Distribution per Year | | |  |  |  |  |  |  |  |  |  |
| 2009 | 410 | (83.0) | 44 | (8.9) | 23 | (4.7) | 12 | (2.4) | 5 | (1.0) | 494 |
| 2010 | 392 | (81.3) | 47 | (9.8) | 22 | (4.6) | 16 | (3.3) | 5 | (1.0) | 482 |
| 2011 | 397 | (80.5) | 49 | (9.9) | 27 | (5.5) | 14 | (2.8) | 6 | (1.2) | 493 |
| 2012 | 398 | (79.1) | 60 | (11.9) | 23 | (4.6) | 16 | (3.2) | 6 | (1.2) | 503 |
| 2013 | 428 | (82.2) | 50 | (9.6) | 22 | (4.2) | 16 | (3.1) | 5 | (1.0) | 521 |
| 2014 | 396 | (80.3) | 53 | (10.8) | 24 | (4.9) | 13 | (2.6) | 7 | (1.4) | 493 |
| 2015 | 402 | (81.1) | 48 | (9.7) | 27 | (5.4) | 15 | (3.0) | 4 | (0.8) | 496 |
|  |  |  |  |  |  |  |  |  |  |  |  |
| Data are in n and Percentage of Annual Total. | | | | | | | | | | |  |
