# Supplementary material for: In-hospital mortality and failure to rescue following hepatobiliary surgery in Germany - a nationwide analysis
Source: BMC Surg. 2020 Jul 29;20:171. doi: 10.1186/s12893-020-00817-5 (PMC7388497; doi:10.1186/s12893-020-00817-5)
Supplement: Supplementary file 8 — Additional file 8: Supplemental file 8. In-hospital Mortality According to Type of Hepatobiliary Resection and Hospital Volume Categories. [file 12893_2020_817_MOESM8_ESM.docx]

| **Supplemental File 8. In-hospital Mortality According to Types of Hepatobiliary Resections and Hospital Volume Categories.** | | | | | | | |
| --- | --- | --- | --- | --- | --- | --- | --- |
|  | |  | **Hospital Volume Categories** | | | | |
|  |  |  | Very Low (1-10) | Low (11-20) | Medium (21-40) | High (41-100) | Very High (>100) |
| Type of Hepatobiliary Resection | | |  |  |  |  |  |
| Trisectionectomy | n/N | | 87/463 | 77/393 | 83/515 | 144/989 | 138/926 |
|  | Obs Rate % | | 18.79 | 19.59 | 16.12 | 14.56 | 14.9 |
|  |  | |  |  |  |  |  |
| Hemihepatectomy | n/N | | 401/3745 | 192/2087 | 191/2131 | 262/3410 | 211/2614 |
|  | Obs Rate % | | 10.71 | 9.20 | 8.96 | 7.68 | 8.07 |
|  |  | |  |  |  |  |  |
| Multiple Segment | n/N | | 151/3308 | 53/1517 | 34/1186 | 63/1272 | 38/1035 |
|  | Obs Rate % | | 4.56 | 3.49 | 2.87 | 4.95 | 3.67 |
|  |  | |  |  |  |  |  |
| Bisegmentectomy | n/N | | 96/2218 | 29/956 | 26/816 | 30/884 | 19/649 |
|  | Obs Rate % | | 4.33 | 3.03 | 3.19 | 3.39 | 2.93 |
|  |  | |  |  |  |  |  |
| Data are in n/N. Observed Rates of Mortality (Obs Rate) in %. | | | | | | | |
